# Optimization of Boron Nitride Sphere Loading in Epoxy: Enhanced Thermal Conductivity and Excellent Electrical Insulation

**DOI:** 10.3390/polym11081335

**Published:** 2019-08-12

**Authors:** Hua Zhang, Rongjin Huang, Yong Li, Hongbo Li, Zhixiong Wu, Jianjun Huang, Bin Yu, Xiang Gao, Jiangang Li, Laifeng Li

**Affiliations:** 1 Advanced Energy Research Center, Shenzhen University, Shenzhen 518060, China; 2 Key Laboratory of Optoelectronic Devices and System of Ministry of Education and Guangdong Province, College of Optoelectronic Engineering, Shenzhen University, Shenzhen 518060, China; 3 Key Laboratory of Cryogenics, Technical Institute of Physics and Chemistry, Chinese Academy of Sciences, Beijing 100190, China; 4 Center of Materials Science and Optoelectronics Engineering, University of Chinese Academy of Sciences, Beijing 100049, China; 5 Beijing Key Laboratory of Construction-Tailorable Advanced Functional Materials and Green Applications, School of Materials Science & Engineering, Beijing institute of Technology, Beijing 100081, China

**Keywords:** boron nitride, isotropous thermal conductivity, epoxy, electrical insulation

## Abstract

Thermally conductive but electrically insulating materials are highly desirable for thermal management applications in electrical encapsulation and future energy fields, for instance, superconducting magnet insulation in nuclear fusion systems. However, the traditional approaches usually suffer from inefficient and anisotropic enhancement of thermal conductivity or deterioration of electrical insulating property. In this study, using boron nitride sphere (BNS) agglomerated by boron nitride (BN) sheets as fillers, we fabricate a series of epoxy/BNS composites by a new approach, namely gravity-mix, and realize the controllable BNS loading fractions in the wide range of 5–40 wt%. The composites exhibited thermal conductivity of about 765% and enhancement at BNS loading of 40 wt%. The thermal conductivity up to 0.84 W·m^−1^·K^−1^ at 77 K and 1.66 W·m^−1^·K^−1^ at 298 K was observed in preservation of a higher dielectric constant and a lower dielectric loss, as expected, because boron nitride is a naturally dielectric material. It is worth noting that the thermal property was almost isotropous on account of the spherical structure of BNS in epoxy. Meanwhile, the reduction of the coefficient of thermal expansion (CTE) was largely reduced, by up to 42.5% at a temperature range of 77–298 K.

## 1. Introduction

Insulating polymer materials are widely applied in electrical engineering and electron devices, such as high voltage transmission and electric generators, and the device encapsulation [1,2,3,4,5]. In addition, the increasing power density and integration level are setting high requirements in heat dissipation, operating reliability and life span of the equipment [6]. Moreover, according to recent advances, magnetic confinement nuclear fusion has attracted significant attention due to its many advantages over other energy technologies [7,8]. However, controlled nuclear fusion has proven to be very difficult to implement because of the harsh conditions of its operating environment, such as strong electric field and ultralow temperature. In a large fusion system, superconducting magnets and insulating materials are the important components. In order to guarantee the security of the fusion system, efficient heat dissipation and low thermal expansion at cryogenic conditions are required for insulating materials [9,10]. Thus, a thermally conductive but electrically insulating material appears to be highly desirable. Until now, many materials have been tested in fusion reactors, including cyanate ester resin [11,12]. However, higher thermal conductivity than pure epoxy (0.1–0.2 W·m^−1^·K^−1^) and thermal expansion suitable for a superconducting magnet is still under development, in view of extremely critical requirements for the materials used in fusion reactors.

According to recent advances, metallic and carbon materials, such as graphene and carbon nanotubes, have been used to enhance thermal conductivity due to their free electrons, which are much more efficient in heat transfer. Unfortunately, adding metallic or carbon particles into polymers will also cause a substantial increase in the electrical conductivity of the composites [13,14,15]. Moreover, ceramic fillers, such as Al_2_O_3_, AlN, BN, SiC and Si_3_N_4_, with different shapes and sizes, have been studied for thermally conductive and electrically insulating composites, as they lack free electrons, so the heat transfer is predominantly realized through phonons [16,17,18,19,20]. As one of the commonly used matrices of polymer materials, epoxy resins are high-performance thermosetting resins combining many excellent properties, such as high mechanical strength, excellent thermal stability, good electrical insulation, and low cost as well as ease of processing. However, they are amorphous materials with low thermal conductivity, which is undesirable for thermal management [21]. In recent years, many efforts have been devoted to increase the thermal conductivity of filler-matrix composites [22,23,24,25]. Typical methods, including using high-aspect-ratio nanofillers, self-assembly, and 3D interconnected structures, have brought about an anisotropic thermal conductivity or reduced flexibility, which was unfavorable for applications [26,27]. Furthermore, the fabrication processes, such as freeze drying [28], electrospinning [4], hot rolling and hot pressing [29] still face the issue of low production, implying that currently available methods are still under-developed for practical applications. Therefore, a convenient and effective fabricating method for thermal conductive and insulating materials is in urgent need.

On account of their good thermal conductivity, electric insulation properties and chemical inertness, hexagonal-boron nitride (h-BN) platelets and nanosheets have been developed as promising dielectric substrates or fillers loaded into the polymer matrix, and have great potential use in electronic encapsulation, high voltage insulation and superconducting magnets insulating systems [30,31,32]. In addition, h-BN belongs to crystals of a 2D layered structure, and manifest a significant anisotropy in physical properties including thermal conductivity and coefficient of thermal expansion (CTE) [33,34]. Boron nitride spheres aggregated by h-BN sheets retain the advantages of h-BN, such as high thermal conductivity, low thermal expansion coefficient and good electrical insulating property, as well as the additional isotropous properties [35]. Further, the BN fillers aggregated by h-BN sheets more easily form an effective heat flow path than randomly laid BN platelets [36]. In principle, the boron nitride sphere (BNS) developed herein is expected to be an excellent filler to enhance the thermal conductive and electrical properties, while maintaining the isotropic performance for the composites. In addition, the BNS structure has several advantages preferred for filler dispersion, such as a decrease in viscosity and increase in possible filler ratio [35]. To maintain the shape and distribution of the fillers, it is essential to use a facile and adjustable composite fabrication method [36]. In our investigation, we report a facile yet feasible approach to prepare a series of epoxy/BNS composites with different filler loading fractions using a planetary gravity mixer. The fabricated filler/matrix composites cost less and were easier to deal with compared with the aforementioned 3D network, especially when used in quantity. Moreover, the isotropic performance is crucial for the insulating structure of the superconducting magnets in fusion systems. However, to our knowledge, the incorporation of BN in epoxy with high loading and investigation of their performances under cryogenic conditions has not yet been reported.

## 2. Materials and Methods

### 2.1. Materials and Characterizations

BNS powders were purchased from Shanghai Bestry Performance Materials Co. Ltd., Shanghai, China. The liquid epoxy resin, NPEL 128 with epoxy value 0.54 eq/100 g, curing agent polyether amine (PEA) D230 and the accelerant tris(dimethylaminomethyl)phenol (DMP-30) were purchased from Dalian Liansheng Trading Co. Ltd., Dalian, China. The thixotropic agent polyamide wax was purchased from ELEMENTIS Co. Ltd., London, UK. The morphology of BNS and the epoxy/BNS composites with different loading fractions were observed with a Hitachi S-4300 scanning electron microscope (SEM) manufactured by Hitachi, Ltd., Tokyo, Japan. Raman spectroscopy (in Via-Reflex) was used to characterize the BNS powders. The particle size distribution of BNS was determined by a laser diffraction particle size analyzer with a LS13320 manufactured by BECKMAN COULTER. Thermal conductivity (λ) was measured using the steady state method over the temperature range from 77 to 298 K and calculated by the following equation:

(1) $$\lambda =Q\frac{L}{\Delta T\times S}$$

where Q is the power supplied, L is the distance of the sample, *S* is the section area of the sample, and Δ*T* is the measured temperature difference. Dielectric constant (ε) and dielectric loss (tan δ) were measured by a TH26008A test fixture. Linear thermal expansion (ΔL/L) was measured through the strain gauge technique over the temperature range from 77 to 298 K and the coefficient of thermal expansion (CTE) was calculated as the slope of the ΔL/L—Temperature curve. DSC (differential scanning calorimetry) was performed on the NETZSCH 404 F3 Pegasus (NETZSCH (Shanghai) Machinery and Instruments Co., Ltd., Shanghai, China) with a heating rate of 5 °C/min under the atmosphere of argon.

### 2.2. Fabrication of Epoxy/BNS Composites

**Scheme 1:**
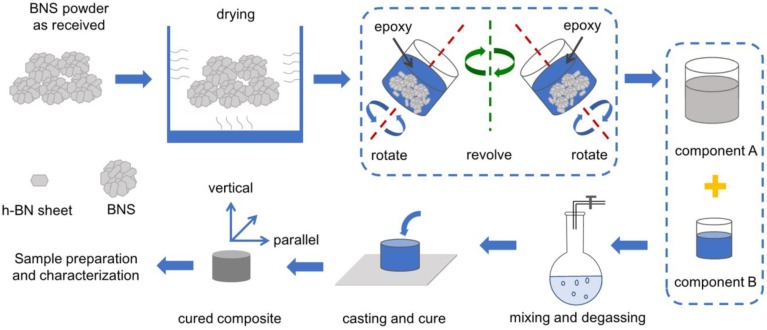
The fabrication process of epoxy/BNS (boron nitride sphere) composites.

Scheme 1 indicates the fabricating process of epoxy/BNS composites. Firstly, the as-received BNS powders were put into an air-dry oven at 100 °C for 24 h to remove superficial water. The thixotropic agent polyamide was dispersed in NPEL 128 to obtain the epoxy matrix. Then, the dried BNS was dispersed in the prepared epoxy matrix via gravity mixing method under vacuum to get component A. Compared with other preparation methods, such as mechanical agitation and magnetic stirring, the gravity mixing method could reduce machine abrasion. There were two polytetrafluoroethylene jars filled with the epoxy mixture equal in quality. Then the jars rotated and revolved at the same time to achieve uniformly filler distribution. After optimizing the mixing procedure via adjusting mixing speed and time, the homogeneous epoxy component A was obtained facilely in a large fabrication capacity. The curing agent D230 and the accelerant DMP-30 were mixed via magnetic stirring according to a mass ratio of 100:3 to get component B. In need of good toughness of the composites, we chose one aliphatic amine D230 as the curing agent because of its flexible molecular chain and rotatable C–O bond. After the component A and B were mixed at a definite weight ratio, the mixture was degassed under vacuum at 40 °C for 20 min. Then the epoxy/BNS mixture was cast into the preheated mold with a release agent. Subsequently, it was put into a vacuum oven to cure the composites, followed by a curing step pre-curing at 40 °C for 6 h and post-curing at 75 °C for 1h. Due to the shear force between BNS and epoxy in the gravity mixing process, some h-BN sheets on the surface of BNS would be peeled off and BNS spheres with different diameters appeared as shown in SEM images of the composites.

## 3. Results and Discussion

### 3.1. Fundamental Physical Properties and Morphology Analysis

**Figure 1:**
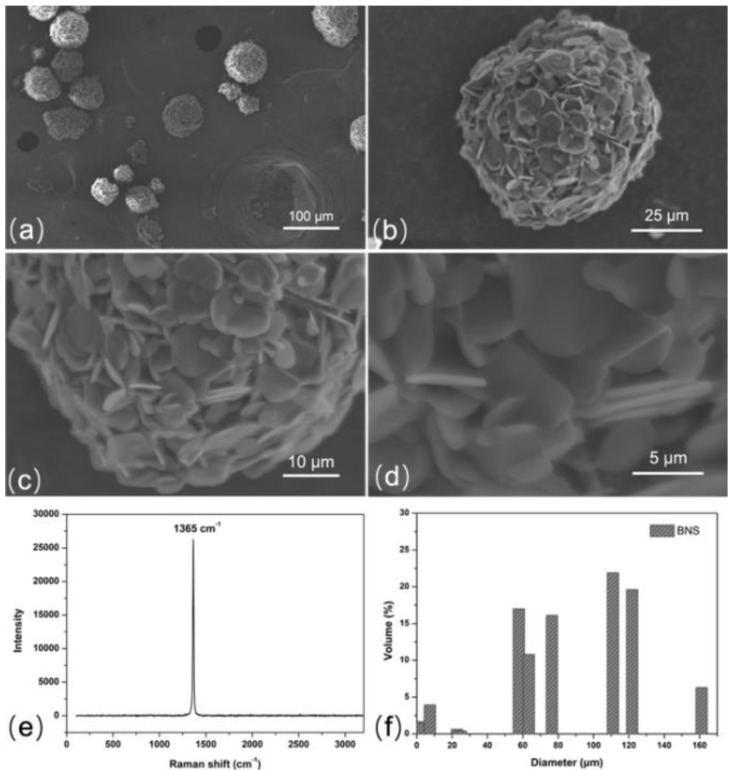
(**a**–**d**) SEM images, (**e**) Raman spectra, (**f**) particle size distribution of the BNS powder.

A laser diffraction method was utilized to examine the particle size distribution of BNS powders as shown in Figure 1f. Diameters of a large proportion of powders were in the range of 60–120 μm. This is a typical feature of BNS, prepared from BN platelets via the granulation method. It is speculated that the broad particle-size distribution is favorable for the improvement of the flow ability and filler packing in the composite materials. Mai et al. carried out research using binary fillers, including isotropic Al_2_O_3_ and anisotropic BN, and better heat transfer pathway was achieved [37]. In our investigation, the BNS was expected to act as an isotropic filler to prepare composites with isotropic properties. The bulk density (ρ) of the cured epoxy/BNS composites was measured based on Archimedes’ principle. Compared with composites using Al_2_O_3_ (ρ = 3.9 g/cm^3^) as fillers, the BNS-based composites exhibited lower density due to low density of BN (ρ = 2.25 g/cm^3^), which is beneficial to reduce the equipment weight. The volume loading fraction of BNS was calculated from the density of each ingredient. The typical data combined with the thermal conductivity values are summarized in Table 1.

Figure 1a–d is the scanning electron microscopy (SEM) images of BNS. The BNS shows a spherical structure with different diameter, which agreed well with the result of a laser diffraction analyzer. It should be noted that the BNS is actually an agglomeration of many h-BN sheets distributed in random directions. The lateral size of h-BN sheets was about 8 μm on average. We do not observe any evident peaks in Raman spectra (Figure 1e), except the band at 1365 cm^−1^, which is the typical BN characteristic and can be attributed to E_2g_ tangential mode, indicating the high purity of BN.

**Figure 2:**
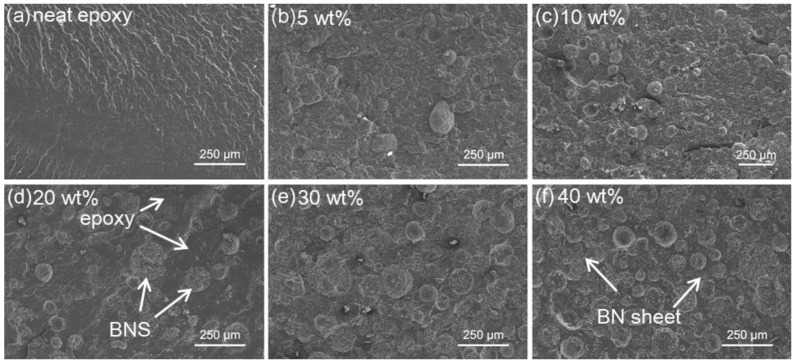
Cross-sectional SEM images of the epoxy/BNS composites with different BNS loading fractions.

**Figure 3:**
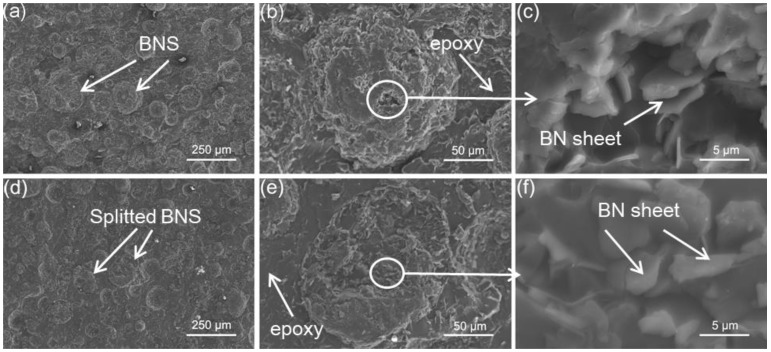
The representative SEM image of the BNS sphere and inside BN sheets loaded in epoxy.

After curing, the cross-sectional structure of the epoxy/BNS composites was analyzed via SEM. The BNS was uniformly distributed in the epoxy matrix as presented in Figure 2. It is notable that at lower loading fractions, the BNS parts were well-separated from each other (see Figure 2b,c) so that the thermal conductivity and CTE did not show an obvious increase or decrease for 5 and 10 wt% samples compared with the neat epoxy. As loading fractions increased, there was more sphere BNS observed from the same field of view as shown in Figure 2d–f. Fillers loaded in the composites also exhibited a sheet structure and were well steeped in epoxy resin. As observed from the cross-sectional images, the diameter of fillers was distributed in a broader range of 50–200 μm because some of the h-BN sheets were peeled off the agglomerates, resulting in a smaller sphere, and the peeled sheets agglomerated to the other, so a larger sphere was obtained. This was the result of blending and shearing forces during the mixing procedure. The diameter distribution in a wide range might enhance the possibility of contact with nearby fillers, as smaller particles fill in the blanks between larger ones, thus forming efficient thermal pathways [22]. To reveal the morphology of BNS loaded in epoxy, we acquired magnified SEM images. As shown in Figure 3, it was demonstrated that the BN sheets inside the spherical agglomerates were in good contact and closer to each other, which was likely to provide reduced interfacial thermal resistance between BN and epoxy (see Figure 3c,f). There were also split BNS spheres (see Figure 3d) on the fractured surface, evidence of the strong interfacial binding force between BNS and the epoxy matrix. The well-dispersed structure may be favorable for large-scale fabrication of good thermal management composite materials.

### 3.2. Thermal Conductivity

**Figure 4:**
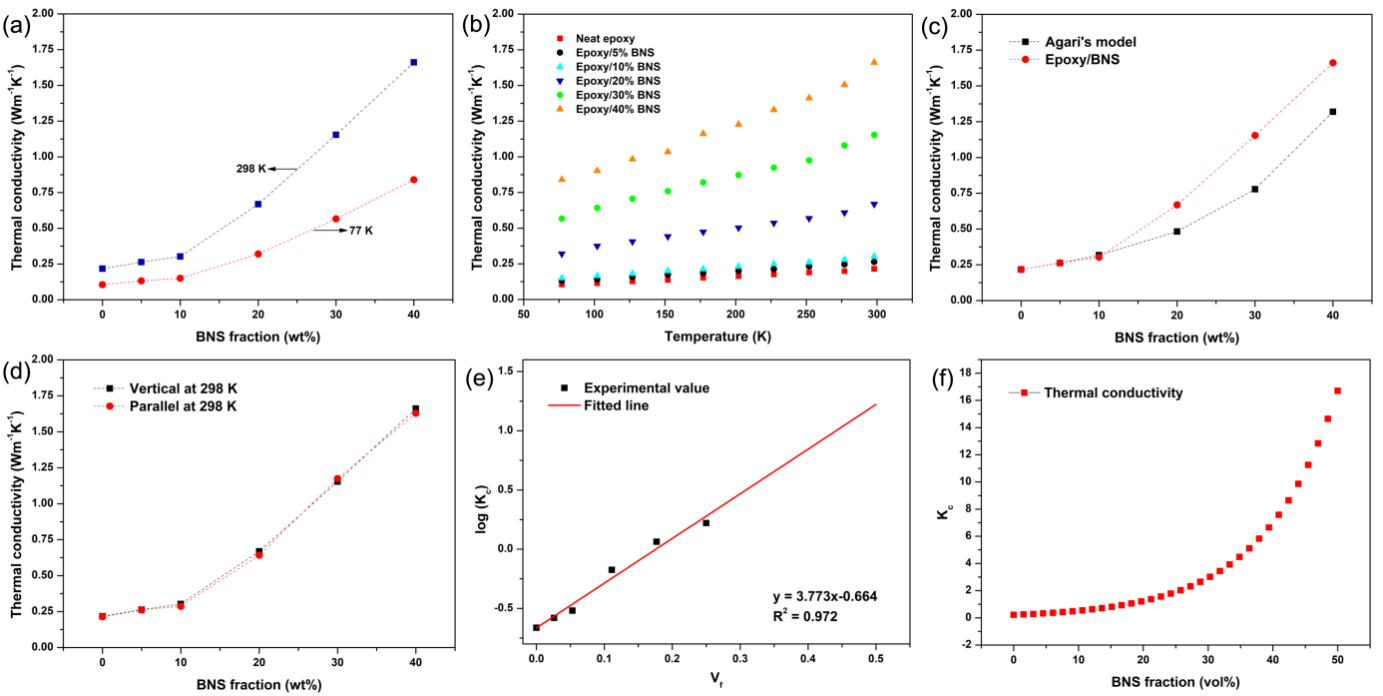
(**a**) Thermal conductivity of epoxy/BNS composites at 77 and 298 K. (**b**) Temperature-dependent thermal conductivity of epoxy/BNS composites. (**c**) Calculated thermal conductivity according to Agari’s model (*C_1_* = 1, *C_2_* = 1) and measured values at 298 K. (**d**) Thermal conductivities from vertical and parallel curing directions of the composites at 298 K. (**e**) Experimental thermal conductivity and fitted line as a function of *V_f_* (volume fraction of fillers). (**f**) Thermal conductivity from fitted results.

In order to evaluate the thermal property of the prepared composites, the thermal conductivity of the epoxy/BNS composites with different BNS loading fractions was measured and calculated as shown in Figure 4. For applications in superconducting magnet insulating systems under cryogenic conditions, thermal conductivity measurements were carried out from 77 to 298 K. As shown in Figure 4a, the neat epoxy resin exhibits a relatively poor thermal conductivity of 0.10 W·m^−1^·K^−1^ at 77 K and 0.21 W·m^−1^·K^−1^ at 298 K because of its amorphous nature. For fractions of 5 and 10 wt%, a slight enhancement of the thermal conductivity was obtained, of 0.26 and 0.30 W·m^−1^·K^−1^ at 298 K, 0.13 and 0.15 W·m^−1^·K^−1^ at 77 K. This phenomenon could be explained as the BNS are not effectively connected and thus thermal pathways are nearly constructed, as we can see from the SEM image of the cured sample in Figure 2. With the loading fraction increased to 20 wt % (and 30 and 40 wt %), the thermal conductivity curves show a rapid ascent. Finally, thermal conductivity reached 1.66 W·m^−1^·K^−1^ at 298 K and 0.84 W·m^−1^·K^−1^ at 77 K under 40 wt% BNS loading, which was improved by approximately eight times, compared to the pure epoxy resin. The measured thermal conductivity confirmed that efficient thermal pathways were well created when the fraction of BNS reached 20 wt % and above. As shown in Figure 4b, the thermal conductivities of the samples increase as the test temperature ranges from 77 to 298 K, as thermal conduction mainly takes place via lattice vibrations forming the phonon flow [38]. As the temperature rises, generation of excited long wavelength phonons accelerates and therefore leads to a higher collision frequency, resulting in the rapid thermal conductivity enhancement. As expected and shown in Figure 4a, the thermal conductivity was increased to 1 W·m^−1^·K^−1^ when the loading fraction was 25 wt%. This result is fairly inspiring, considering that 40 wt% AlN and 60 wt% h-BN sheets were introduced to obtain the comparable effect [39,40].

To reveal the relationship between loading fraction and the thermal conductivity, and then to make effective predictions under high loadings, theoretical calculations were carried out. The thermal conductivity of composites can be predicted with various models in which Agari’s equation [41] is a classic as shown in the following:

(2) $$\log k_{c}=V_{f}\cdot C_{2}\cdot \log k_{f}+\left(1-V_{f}\right)\cdot \log\left(C_{1}\cdot k_{p}\right)$$

where $k_{p}$ and $k_{f}$ are the thermal conductivities of polymer matrix and filler, respectively. In this work, they are 0.217 and 300 W·m^−1^·K^−1^ [22,33]. $V_{f}$ is the volume fraction of the filler calculated according to the weight loading fractions and the bulk density of BN (Table 1). $C_{1}$ is related to the thermal conductivity change of the polymer matrix measuring the impact of fillers on the secondary structure of the polymer matrix, such as the degree of crystallinity and crystal size. $C_{2}$ is related to the capability of the particles to easily form conductive chains through the material and generally was 0–1. According to previous research [41,42], firstly $C_{1}$ could be considered to be 1 in this work. At the same time, by setting $C_{2}$ as 1, the calculated value according to Agari’s model is shown in Figure 4c. One can see that the experimental value was slightly lower at 5 and 10 wt % but much higher at 20, 30 and 40 wt % than the calculated values. According to the experimental value in this work, the logarithmic plot of thermal conductivity with respect to the filler content ($V_{f}$) is presented in Figure 4e, and parameters $C_{1}$ and $C_{2}$ are calculated to be about 1 and 1.79. The value of $C_{1}$ confirmed that the introduction of BN has nearly no effect on the crystalline structure of the polymer matrix. The higher $C_{2}$ value indicates more ease in forming conductive pathways [43,44]. It could be concluded that the randomly dispersed BNS structure agglomerated by platelet h-BN is favorable to form thermally conductive pathways at 20 wt % or higher loadings. Finally, these pathways would form the network to improve the thermal conductivity of the epoxy composites. The $k_{c}$ calculated from the fitted logarithmic plot as a function of loading fraction (vol %) demonstrated that the thermal conductivity is increased with rising BNS fractions, as shown in Figure 4f. In other words, if the matrix was well-optimized by reducing the viscosity, higher filler content and consequent high thermal conductivity could be achieved.

As the loaded BNS were spherical structures, the cured epoxy composite shows nearly the same thermal conductivity in the parallel and vertical directions (Scheme 1) to the curing plane, as shown in Figure 4d. Platelet-shaped fillers have a natural tendency to align parallel with one another, especially at high loading levels, leading to anisotropic thermal conductivity of cured composites [45,46]. For practical applications, the convenience of preparation and performance uniformity, especially for thermal properties, was the important aspect for a thermally conductive and electrically insulating adhesive. In this work, we use BNS as fillers and apply this simple yet efficient approach to settle this problem.

### 3.3. Linear Thermal Expansion

**Figure 5:**
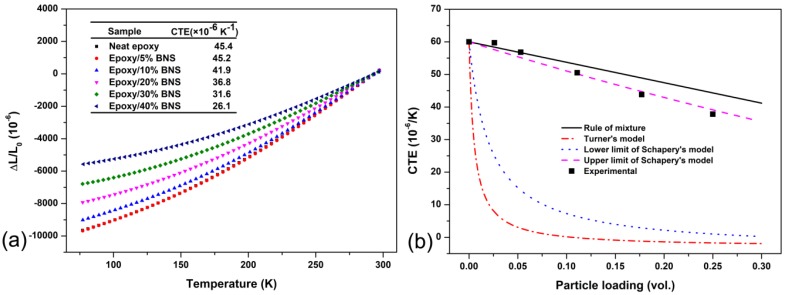
(**a**) Linear thermal expansion curves of epoxy/BNS composites in temperature range of 77–298 K at a heating rate of 5 Kmin^−1^; (**b**) Measured CTEs and calculated CTEs by analytical models.

For thermal management applications, especially in superconducting magnet systems, CTE is another key parameter used to evaluate the performance of insulating materials. The unmatched CTE of epoxy resin and conductor may induce thermal stress as thermal cycling then causes insulating layer debonding, which will significantly affect the reliability of magnet systems. Hence, reducing the CTE of insulating materials is necessary. Introducing dimension stable inorganic filler to the polymeric matrix is a typical method [10]. The linear thermal expansion of the neat epoxy and the epoxy/BNS composites in parallel directions with different loading fractions as a function of temperature ranging from 77 to 298 K is shown in Figure 5a, where the CTE values are calculated by the slope of the curves as shown in the inset. The CTE was determined based on the following equation:

(3) $$\alpha =\frac{1}{L_{0}}\frac{dL}{dT}$$

where $L_{0}$ is the initial length of the sample.

With the increase of BNS content, a gradual CTE reduction was observed. The CTE value of the pure epoxy resin is 45.4 × 10^−6^ K^−1^ whereas it decreased to 26.1 × 10^−6^ K^−1^ when the BNS content reached 40 wt %. Owing to the low CTE of BN, the high loadings of BNS in composites may result in the restriction of the polymer chain movements, giving rise to the CTE reduction of the epoxy composites. The lower CTE of the insulating materials will effectively minimize the mismatch thermal expansion, thus contribute a lot during thermal cycling making the magnetic system more reliable.

In order to confirm the experimental results, we compared measured the CTE value at 298 K with the calculated value according to several models as shown in Figure 5b. Typically, three models are used to predict the CTE of polymer/filler composites. Firstly, the rule of mixtures is represented by Equation (4):

(4) $$\alpha_{c}=\alpha_{m}\left(1-\varphi_{f}\right)+\alpha_{f}\varphi_{f}$$

where $\varphi_{f}$ is the volume fraction of filler, $\alpha_{f}$ is the CTE of the filler (−2.7 × 10^−6^ K^−1^) [47], $\alpha_{m}$ is the CTE of the matrix (60 × 10^−6^ K^−1^, Figure S1). We can see from Figure 5b that the rule of mixtures overpredicted the CTEs compared with the experimental values, indicating the existence of interfacial interactions between the filler and the matrix. Secondly, Turner’s model is usually applied to account for the filler–matrix interactions, incorporating the bulk modulus of both filler ($K_{f}$ = 450 GPa) and matrix ($K_{m}$ = 2.4 GPa) [48] into Equation (5). However, it failed to accurately predict the CTE of the composites as it is appropriately applied only to unidirectional, fiber-reinforced composites [49]:

(5) $$\alpha_{c}=\frac{\left(1-\varphi_{f}\right)K_{m}\alpha_{m}+\varphi_{f}K_{f}\alpha_{f}}{\left(1-\varphi_{f}\right)K_{m}+\varphi_{f}K_{f}}$$

Thirdly, Schapery’s model includes a determination of the upper ($\alpha_{c}^{u}$) and lower limit ($\alpha_{c}^{l}$) values for the composites, as shown in Equations (6) and (7):

(6) $$\alpha_{c}^{u}=\alpha_{m}+\frac{K_{f}}{K_{c}^{l}}\frac{\left(K_{m}-K_{c}^{l}\right)\left(\alpha_{f}-\alpha_{m}\right)}{\left(K_{m}-K_{f}\right)}$$

(7) $$\alpha_{c}^{l}=\alpha_{m}+\frac{K_{f}}{K_{c}^{u}}\frac{\left(K_{m}-K_{c}^{u}\right)\left(\alpha_{f}-\alpha_{m}\right)}{\left(K_{m}-K_{f}\right)}$$

where $K_{c}^{u}$ and $K_{c}^{l}$ are determined using Equations (8) and (9):

(8) $$K_{c}^{l}=K_{m}+\frac{\varphi_{f}}{\frac{1}{K_{f}-K_{m}}+\frac{3\left(1-\varphi_{f}\right)}{\left(3K_{m}+4G_{m}\right)}}$$

(9) $$K_{c}^{u}=K_{f}+\frac{1-\varphi_{f}}{\frac{1}{K_{m}-K_{f}}+\frac{3\varphi_{f}}{\left(3K_{f}+4G_{f}\right)}}$$

where $G_{m}$ and $G_{f}$ are the shear moduli of the matrix (0.9 GPa) and the filler (112 GPa) [47], respectively. As shown in Figure 5b, the upper limit of Schapery’s model provided a very close approximation of the CTE values for epoxy/BNS composites and the model may be very useful to make effective predictions for different filler loading.

### 3.4. Dielectric Properties

**Figure 6:**
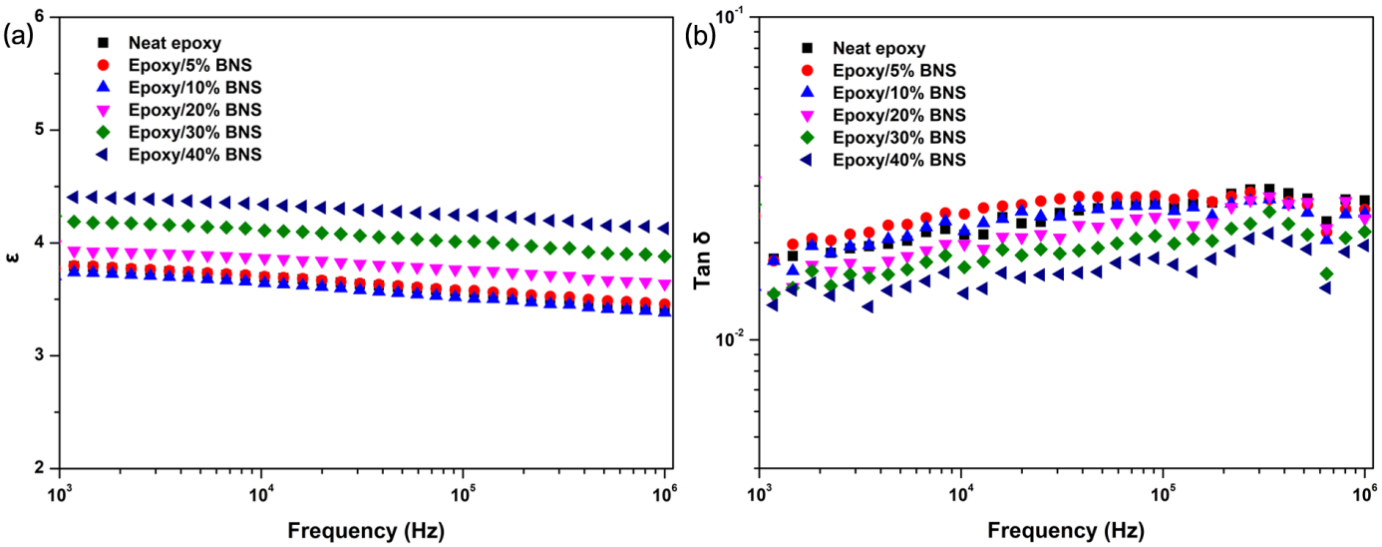
(**a**) Dielectric constant and (**b**) dielectric loss tangent as a function of frequency for the epoxy/BNS composites.

For thermal management in fields of electrical devices and equipment, the dielectric property is required. The effects of BNS and loading fraction on dielectric properties of epoxy/BNS composites were tested and are shown in Figure 6. In Figure 6a, the dielectric constant modestly decreases as the frequency increases, which could illustrate that the dipole moment rotation in BN gradually lagged when the frequency of the electric field was increased. The dielectric constant also increases with increased BNS loadings, which is attributed to the larger number of dipole moment in the composites. Further, as the filler content increases, the concentration of BNS reaches the region to contact with each other, and a continuous channel can be formed by tunneling effects and electron transitions [50]. Therefore, as the polarization performance of the composites improves, the dielectric constant increases. In detail, the dielectric constant of epoxy/BNS composites with 40 wt % filler loading reached 4.13 at 1 MHz. On the other hand, as the loading fractions rose, the dielectric loss decreased to 0.02 at 1 MHz at 40 wt % BNS loading, as shown in Figure 6b. Because of the formed channel along with the increasing loading fraction, the effects of interfacial gaps and defects may weaken, and the dielectric loss slightly decreases [51].

### 3.5. DSC Measurements

**Figure 7:**
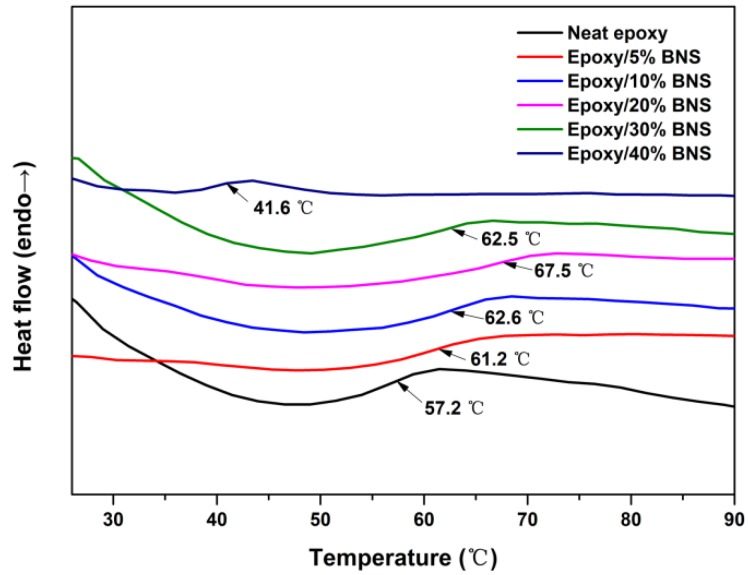
DSC (differential scanning calorimetry) curves of epoxy/BNS composites with a heating rate of 5 °C min^−1^ under Ar.

The glass transition of polymer is a molecular relaxation involving cooperative segmental motion, the rate of which will depend on temperature [52]. Figure 7 demonstrates that at lower loading fractions, such as 5 and 10 wt %, T_g_ shifted towards a higher temperature due to the good interactions between BNS and epoxy resin restricting the mobility of the epoxy chain, as reported earlier [53]. The lower T_g_ of the higher loading composites, including 30 and 40 wt % fractions, is attributed to the numerous BNS that may destroy part of the cross-linked epoxy cured system. After all, T_g_ was lower than in the normally used epoxy composites, resulting from the flexible curing agent polyether amine D230, in which the C–O bond could not be totally frozen at low temperature, thus improving the toughness of the composites under cryogenic conditions [54].

## 4. Conclusions

Epoxy/BNS composites with superior comprehensive performance of high isotropous thermal conductivity, good electrical insulation, and low coefficient of thermal expansion have been successfully prepared. In particular, a convenient preparation method using a gravity mixer brought about large-scale production, and the resulting isotropous thermal conductivity makes the composite more applicable. At a BNS loading fraction of 40 wt %, the composite exhibited a 765% thermal conductivity enhancement compared with neat epoxy, reaching 1.66 W·m^−1^·K^−1^ at 298 K and 0.84 W·m^−1^·K^−1^ at 77 K, and a 42.5% reduction in CTE to 26.1 × 10^−6^ K^−1^. More importantly, the higher dielectric constant and lower dielectric loss indicates that this filler type was sufficiently favorable. We successfully addressed the main problems of obtaining high thermal conductivity and high electrical insulation at the same time, and also met the challenge of low CTE and inhomogeneous thermal performances when a large quantity of materials was required. The present work may promote the application process of thermal management materials in fields of electron device encapsulation and superconducting magnet insulation systems.

## Figures and Tables

**Table 1:** The volume fraction of filler and the density and thermal conductivities of the composites.

BNS (wt %)	BNS (vol %)	ρ(gcm^−1^) ^1^	Calculated (W·m^−1^·K^−1^) ^2^	Experimental (W·m^−1^·K^−1^) ^3^
0	0	1.133	0.217	0.217
5	2.6	1.139	0.274	0.263
10	5.3	1.142	0.322	0.303
20	11.1	1.289	0.494	0.668
30	17.7	1.314	0.806	1.154
40	25	1.404	1.385	1.661

^1^ The density measured at 298 K by the Archimedes’ principle using deionized water as the medium. ^2^ The thermal conductivity was calculated according to Agari’s model. ^3^ The thermal conductivity was measured using the steady state method.

## Supplementary Materials

The following are available online at https://www.mdpi.com/2073-4360/11/8/1335/s1, Figure S1: Fitted curves of the average CTE for epoxy/BNS composites as a function of temperature.
