# Supplementary material for: Optimization of Boron Nitride Sphere Loading in Epoxy: Enhanced Thermal Conductivity and Excellent Electrical Insulation
Source: Polymers (Basel). 2019 Aug 12;11(8):1335. doi: 10.3390/polym11081335 (PMC6723785; doi:10.3390/polym11081335)
Supplement: Supplementary file 1 [file polymers-11-01335-s001.pdf]

Optimization of Boron Nitride Sphere Loading in Epoxy: Enhanced Thermal Conductivity and Excellent Electrical Insulation

Hua Zhang,[123] Rongjin Huang,*[34] Yong Li,[3] Hongbo Li,[5] Zhixiong Wu,[3] Jianjun Huang,[1] Bin Yu,[2] Xiang Gao,[1] Jiangang Li [1] and Laifeng Li*[34]

[1] Advanced Energy Research Center, Shenzhen University, Shenzhen 518060, China.
[2] Key Laboratory of Optoelectronic Devices and System of Ministry of Education and Guangdong Province, College of Optoelectronic Engineering, Shenzhen University, Shenzhen, 518060.
[3] Key Laboratory of Cryogenics, Technical Institute of Physics and Chemistry, Chinese Academy of Sciences, Beijing 100190, China.
[4] Center of Materials Science and Optoelectronics Engineering, University of Chinese Academy of Sciences, Beijing 100049, China.
[5] Beijing Key Laboratory of Construction-Tailorable Advanced Functional Materials and Green Applications, School of Materials Science & Engineering, Beijing institute of Technology, Beijing 100081, China.
* Corresponding authors
Email: huangrongjin@mail.ipc.ac.cn (R. H.); lfli@mail.ipc.ac.cn (L. L.)

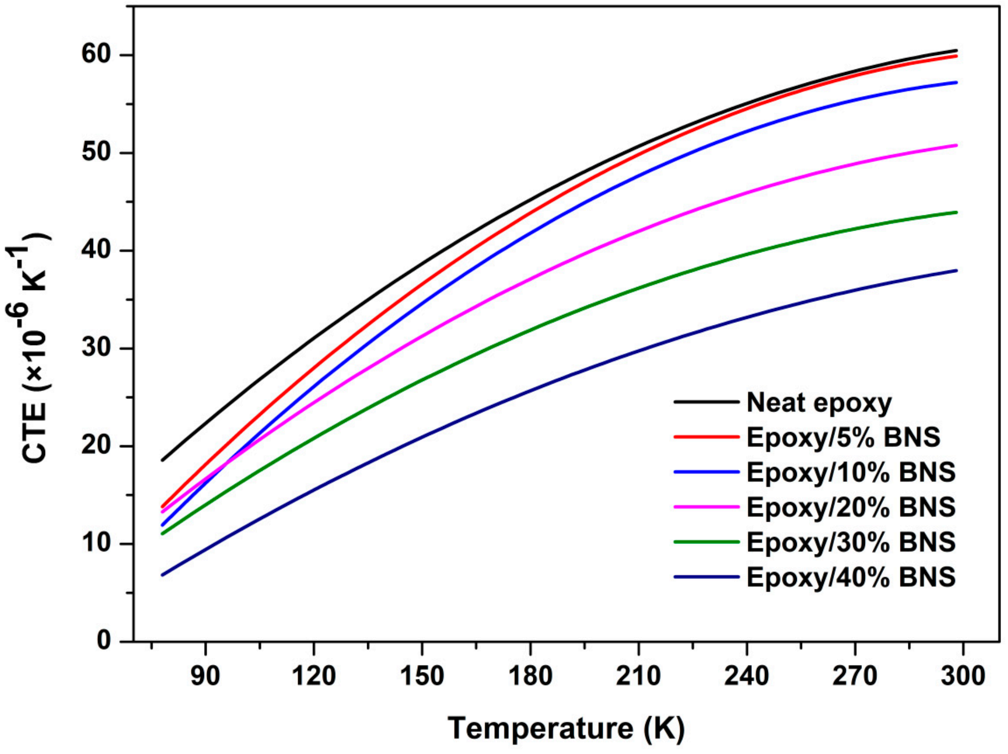

Figure S1. Fitted curves of the average CTE for epoxy/BNS composites as a function of temperature.
